# Guanine nucleotide directed co-assembly with Pt-complexes for tailoring chiroptical properties and multimodal molecular recognition

**DOI:** 10.1039/d5sc07250f

**Published:** 2025-11-14

**Authors:** Jiannan Xiao, Fang Zeng, Zhi-Wang Luo, Bo Yang, Jun Song, Pengfei Duan, Xue Jin, Yong Chen, Zhen-Qiang Yu

**Affiliations:** a College of Chemistry and Environmental Engineering, Shenzhen University Shenzhen 518060 China zqyu@szu.edu.cn; b College of Physics and Optoelectronic Engineering, Shenzhen University Shenzhen 518060 China; c Laboratory of Nanosystem and Hierarchical Fabrication, National Center for Nanoscience and Technology Beijing 100190 China jinx@nanoctr.cn; d Technical Institute of Physics and Chemistry, University of Chinese Academy of Sciences Beijing 100190 China chenyong@mail.ipc.ac.cn

## Abstract

Modulating co-assembly behavior *via* a subtle structural difference in biomolecular partners holds great potential, yet remains underexplored. Herein, we report an in-depth photophysical investigation, revealing that guanine nucleotides (GMP, GDP, and GTP) direct the co-assembly of organoplatinum complex (A), where the phosphate number controls the ribose–Pt(ii) distance and yields distinct supramolecular structures with unique chiroptical properties. For the monophosphate guanine nucleotide (GMP), the close distance between the chiral unit and the Pt(ii) center enables efficient ground-state chiral induction and leads to enhanced circular dichroism (CD). While the diphosphate guanine nucleotide (GDP) adopts partially intercalated edge-associated geometry and enhanced photoluminescence (PL) efficiency by minimizing non-radiative transition through facilitating metallophilic interactions and restricting the rotational freedom. The triphosphate guanine nucleotide (GTP) gives rise to an asymmetrically organized assembly, and the chiral ribose units are completely located outside the molecular layer, which lead to enhanced circularly polarized luminescence (CPL). The multimodal optical outputs enable specific recognition of GMP, GDP, and GTP, respectively, offering a multifunctional strategy for molecular discrimination based on optical multi-channel responses. By bridging the molecular-scale electronic structure, spatial interaction sites, and supramolecular packing, this work highlights the underlying structure–function coupling that governs diverse optical outputs in these nucleotide-directed co-assemblies.

## Abstract 2

Supramolecular co-assembly driven by phosphate variation modulates spatial interaction and supramolecular packing, enabling mode-selective chiroptical responses and precise molecular recognition.

## Graphical abstract

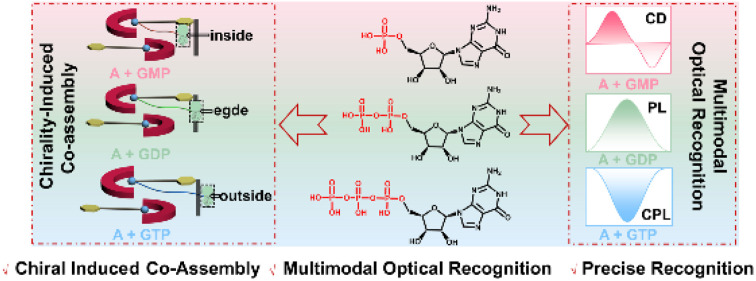

## Introduction

Nucleotides, the fundamental constituents of DNA and RNA, also serve as essential energy donors for life processes such as ATP and GTP, where the number of phosphate groups decisively governs their biological functions.^1^ Yet, discriminating nucleotides with only one or two phosphate group difference (*e.g.*, GMP, GDP, and GTP) remains a major challenge, because their molecular frameworks are nearly identical and conventional analytical techniques lack the ability to specifically characterize the subtle molecular structure difference.^2^ In nucleotides, the number of phosphate groups can regulate electrostatic interaction, coordination interaction, and the number of hydrogen bonds at binding sites, thereby guiding the chiral supramolecular assemblies: changes in the phosphate number can adjust binding geometries and intermolecular forces to yield distinct assembly architectures. Assembly differences may lead to different photoluminescence (PL) behaviors and chirality characteristics, including circular dichroism (CD) and circularly polarized luminescence (CPL), which can be applied to molecular recognition through chiroptical characteristics of the assembled system. Therefore, it is expected to realize nucleotide recognition through nucleotide directed chiral assembly.

Followed by this rationale, we adopt chiral molecule directed co-assembly to amplify the structural differences.^3^ Chirality is pervasive in biology, and many biomolecules possess defined absolute configurations capable of templating supramolecular order.^4^ When a luminophore was co-assembled with a chiral molecule, the chiral molecule can bias the packing geometry, helical structure, and local environments of the co-assembled luminophore. Even slight variations in phosphate number or chiral context can change assembly pathways, which are subsequently amplified into readily resolvable changes in the PL quantum yield and chiroptical readouts (CD, CPL, *etc*.).^4*a*,5^ Amplification addressed the molecular recognition bottleneck of highly similar nucleotides, enabling sensitivity and selectivity discrimination.

Building on this principle, organoplatinum complexes are powerful luminescent materials for chiral co-assembly due to their high luminescence and directed chiral co-assembly properties.^6^ Their rich photophysical properties, driven by complex electronic structures, and square-planar d^8^ centers with strong spin–orbit coupling, enable efficient room-temperature phosphorescence.^7^ A key advantage lies in their highly sensitive metal-to-ligand (MLCT) and metal–metal-to-ligand (MMLCT) charge-transfer transitions, coupled with strong π–π stacking and Pt⋯Pt interactions, which endow organoplatinum complexes with directed chiral co-assembly characteristics.^8^ The above-mentioned features of organoplatinum complexes make them an ideal model for investigating excited-state chirality and hierarchical supramolecular photophysical behavior, offering a distinct advantage in molecular recognition of specific nucleotides by providing highly sensitive and amplified photophysical information.^9^

**Scheme 1:**
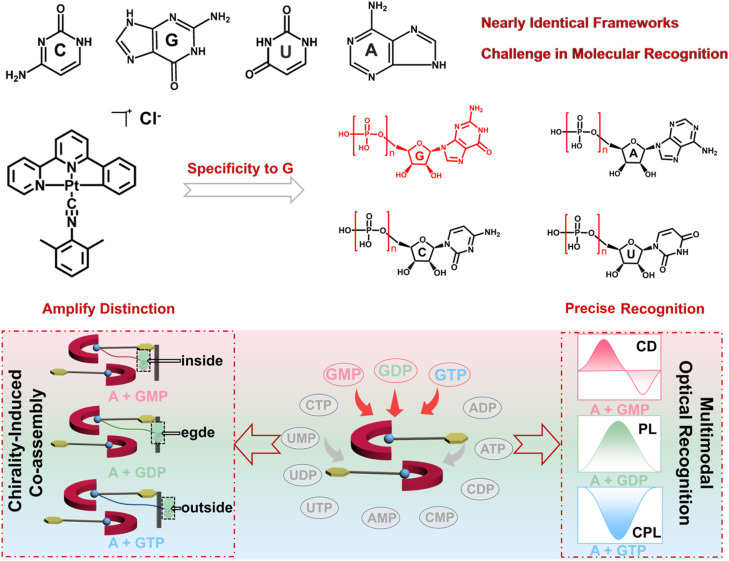
Schematic illustration of chirality-induced supramolecular co-assembly and its multimodal optical recognition. (Top) molecular structures of nucleotides show nearly identical frameworks, highlighting the intrinsic challenge in molecular recognition. (Middle) a chirality-induced co-assembly process driven by the specific recognition between the guanine nucleotide and organoplatinum complex A. (Bottom) schematic illustration of three different co-assemblies between GMP, GDP, or GTP and complex A, highlighting their distinct supramolecular architectures. The co-assembly pathway enables the precise discrimination of three structurally similar nucleotides by multimodal optical output.

In this research, three guanine nucleotides, GMP, GDP, and GTP, were selected to modulate their co-assembly behavior of a phosphorescent organoplatinum complex (complex A), yielding three supramolecular co-assemblies with unique optical or chiroptical properties and enabling multimodal molecular recognition of GMP, GDP, and GTP based on their different CD, PL, and CPL results (Scheme 1). Through comprehensive photophysical analyses and electronic structure calculations, the results elucidate how the number and position of phosphate groups govern molecular packing, Pt⋯Pt distances, and ground- and excited-state chirality induction between guanine nucleotides and complex A. Notably, A + GMP yields the strongest CD signal due to enhanced ground-state chiral induction near the Pt(ii) center; A + GDP exhibits the most intense phosphorescence, driven by partial intercalation of GDP into complex A and optimizes metallophilic interactions; while A + GTP obtains the most pronounced CPL *via* enhanced excited-state chirality. This research constructs a multi-channel molecular recognition model of guanine nucleotides (GMP, GDP, and GTP) with amplified optical or chiroptical responses and reveals how the electronic behavior and supramolecular geometry of co-assemblies affect their optical or chiroptical properties. Besides, the research also paves a versatile platform for designing photofunctional materials with precise control on molecular and supramolecular scales.

## Results and discussion

### Self-assembly behaviour of complex A

The organoplatinum complex A^10^ consists of a tridentate N^N^C ligand and an isocyanide group coordinated to the Pt(ii) center (Fig. S1). The square-planar d^8^ configuration of Pt(ii) facilitates Pt⋯Pt interactions through a d_*z*^2^_ orbital overlap, while the aromatic N^N^C ligand enables π–π stacking. These synergistic interactions promote aggregation in aqueous media *via* hydrophobic effects. As reported in Fig. S2–S4, complex A exhibits a solvent-dependent emission: an ^3^MLCT-based band at ∼550 nm in methanol and a red-shifted ^3^MMLCT emission around 700 nm in water, reflecting the formation of Pt⋯Pt stacked aggregates. The solvent-induced spectral shift highlights the self-assembly behavior of complex A driven by metal–metal and π–π interactions.

As the water content increases in MeOH/H_2_O mixtures (Fig. S5a), a broad absorption band associated with aggregated species appears, which is absent in pure methanol, indicating ground-state aggregation driven by Pt⋯Pt interactions and π–π stacking (MMLCT). Correspondingly, phosphorescence spectra show a solvent-dependent red-shift: in methanol, the emission is ^3^MLCT-dominated, whereas in water-rich solutions, a new intense ^3^MMLCT emission emerges with a higher quantum yield and a longer lifetime (Fig. S5b, c, Table S1 and Fig. S6). The CIE coordinates reflect the color change consistent with optical observations (Fig. S5d), and dynamic light scattering confirms larger aggregate dimensions in aqueous solution (Fig. S7). These results highlight the critical role of solvent polarity in modulating the self-assembly and photophysical behavior of complex A.

The concentration-dependent self-assembly of complex A in aqueous solution was examined by absorption and emission measurements (Fig. S5e, f and S8). Increasing the concentration enhances the MMLCT absorption and emission, reaching a maximum at intermediate concentrations due to strengthened Pt⋯Pt interactions and π–π stacking.^11^ At higher concentrations, excessive aggregation disrupts optimal metal–metal and π–π interactions, leading to reduced electronic coupling and quenched emission. These results indicate that self-assembly and the resulting photophysical behavior of complex A are strongly concentration-dependent.

### Chirality-induced co-assembly and specific recognition of guanine nucleotides

**Fig. 1:**
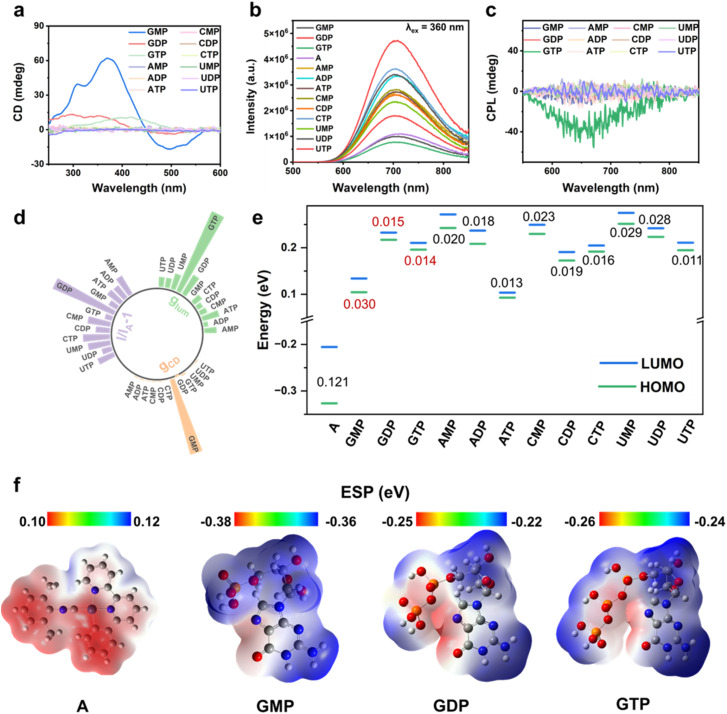
Multimodal optical responses of complex A upon interaction with various nucleotides. (a) CD spectra of complex A in the presence of different nucleotides, revealing distinct ground-state chiral induction effects by GMP. (b) PL spectra showing emission enhancement depending on GDP. (c) CPL spectra of complex A upon co-assembly with various nucleotides, demonstrating enhanced CPL intensities by GTP. (d) Polar plot summarizing the *g*_CD_, emission intensity (*I*/*I*_A_ − 1) at 700 nm, and *g*_lum_, highlighting guanine-induced multimodal optical responses. (e) HOMO–LUMO energy levels of complex A and nucleotides, indicating optimal orbital alignment and complementary energy gaps that facilitate efficient charge transfer between complex A and the guanosine nucleotide. (f) DFT-calculated electrostatic potential (ESP) maps of complex A and guanine nucleotides, revealing charge complementarity that underlies selective co-assembly.

Nucleotides, with inherent chirality and conformational flexibility, serve as ideal chiral templates in supramolecular assembly. Among twelve natural nucleotides, complex A selectively co-assembles with GMP, GDP, and GTP under chiral induction, forming distinct supramolecular structures with diverse optical and chiroptical properties, as revealed by CD, PL, and CPL spectroscopy. Equimolar mixtures (5 × 10^−4^ M) of complex A with each nucleotide further confirmed this selectivity, showing pronounced optical responses only for the guanine-containing species, highlighting their unique selectivity for complex A. As shown in Fig. 1a, CD spectroscopy revealed a strong CD signal for A + GMP, characterized by a prominent negative Cotton effect at ∼480 nm and the most intense positive signal at ∼380 nm, according to the MMLCT- and MLCT-induced charge transfer transition. In comparison, other monophosphate nucleotides exhibited significantly weaker CD signals, indicating specific CD response of complex A to GMP (Fig. S9). Moreover, the ground-state chirality of the solid assemblies was assessed using diffuse reflectance circular dichroism (DRCD) spectroscopy (Fig. S10). The DRCD spectra reveal distinct chiral responses among the different assembled powders. Notably, the A + GMP complex exhibits a pronounced positive DRCD signal around 340 nm, indicating a strong ground-state chiral induction that is not caused by light scattering effects. Meanwhile, fluorescence detected circular dichroism (FDCD) spectra reveal prominent chiral signals upon nucleotide binding (Fig. S11). While complex A alone shows negligible signals, the A + GMP assembly displays a pronounced positive band at 280–350 nm, indicating strong chiral induction. In contrast, A + GDP and A + GTP remain nearly silent, suggesting weaker chirality transfer under identical conditions. These observations highlight the pivotal role of GMP in promoting a robust ground-state chiral environment within the assembly.

PL measurements revealed that GDP induced the most pronounced phosphorescence enhancement at ∼670 nm, along with the highest quantum yield, extended excited-state lifetime, and exceptional selectivity among all nucleotides (Fig. 1b, S12–S15 and Table S1). CPL spectroscopy offered an additional detection channel, with GTP inducing a distinct CPL signal at 680 nm—significantly stronger than the negligible responses from other nucleotides (Fig. 1c and S16). The relative |*g*_CD_| values, emission intensities (*I*/*I*_A_ − 1), and *g*_lum_ values (Fig. 1d and S17) further emphasize the strong and selective responses of GMP, GDP, and GTP across multiple optical outputs. These distinct chiroptical responses enable multimodal molecular recognition, offering a robust strategy for precise analyte identification based on signal-channel specificity.

DFT calculations were conducted to probe the molecular energy levels underlying complex A's selective co-assembly with guanine-based nucleotides. As shown in Fig. 1e, f and S18, the distinct chiroptical responses—CD for GMP, PL for GDP, and CPL for GTP—arise from a synergistic match of electrostatic complementarity and electronic energy alignment, a feature not observed with other nucleotides.^12^ Complex A, featuring a positive electrostatic potential and low-lying HOMO/LUMO levels, preferentially engages with the negatively charged phosphate groups and electron-rich guanine sites (N and O of guanosine) of G-series nucleotides. The close energy-level alignment between GMP and complex A, combined with electrostatic complementarity, drives the formation of a unique supramolecular architecture through electrostatic interactions. In contrast, A + GDP and A + GTP benefit from stronger phosphate-driven interactions and minimal energy gaps (0.015 and 0.014 eV *vs.* 0.121 eV for A), promoting efficient energy transfer. Despite similar energy gaps (*e.g.*, ATP, UTP, *etc*.), other nucleotides show insufficient electrostatic interaction and energy-level compatibility with complex A, resulting in inefficient electronic transitions and limited supramolecular co-assembly.

### Elucidation of interaction modes between guanine nucleotides and complex A

The morphological characteristics of the assemblies formed by complex A with different guanine nucleotides were thoroughly investigated using scanning electron microscopy (SEM). Samples were prepared by depositing onto silicon wafers, followed by solvent evaporation. As shown in Fig. S19A, the SEM image of complex A in water reveals a self-assembled structure composed of uniformly aligned nanowires. Upon interaction with GMP, the SEM image (Fig. S19B) exhibits a transformation from linear nanowires to slender helical structures that further aggregate into clusters—these helices serve as the origin of chirality in the supramolecular system. In contrast, the A + GDP mixture (Fig. S19C) shows a network of interwoven nanofibers forming a highly interconnected architecture. The A + GTP mixture (Fig. S19D) exhibits petal-like helices with significantly larger dimensions than those in both A + GMP and A + GDP systems, consistent with dynamic light scattering (DLS) data (Fig. S20), indicating the formation of larger aggregates. These distinct morphologies result from different coordination modes and molecular lengths of the nucleotides, which govern the extent of π–π stacking and Pt⋯Pt interactions. Consequently, the helical architectures of the GMP and GTP assemblies lead to stronger chiroptical responses (CD and CPL), whereas the networked GTP structure, with partial disorder, shows weaker optical activity and lower emission intensity. These structural and dimensional variations suggest different interaction mechanisms between complex A and the three guanine nucleotides.

**Fig. 2:**
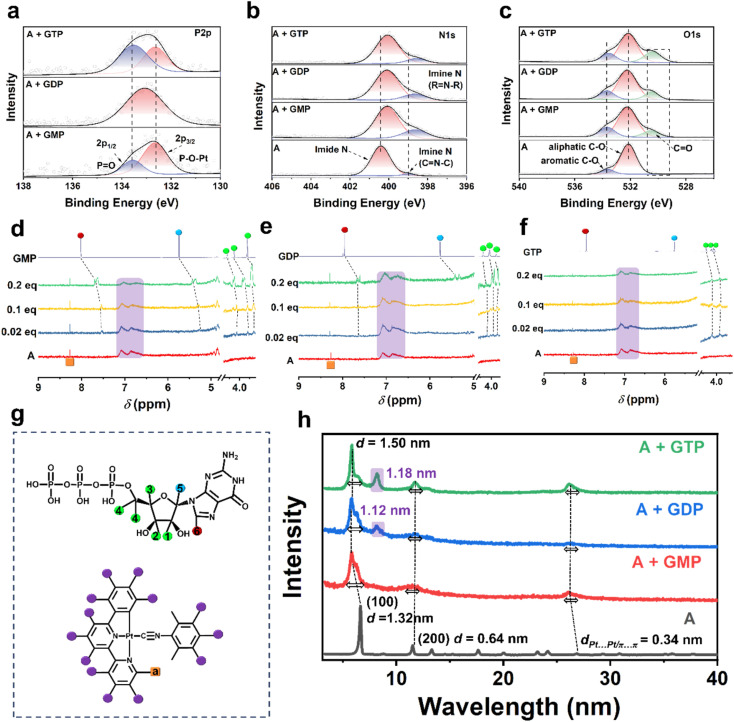
Characterization of molecular interactions governing the co-assembly of complex A with guanosine nucleotides. (a–c) High-resolution XPS spectra show the binding energy change in (a) P 2p, (b) N 1s, and (c) O 1s regions of complex A before and after co-assembly with different guanosine nucleotides (GMP, GDP, and GTP). (d–f) Partial ^1^H NMR spectra (700 MHz, 298 K) of complex A (0.5 mM in D_2_O) upon stepwise addition of (d) GMP, (e) GDP, and (f) GTP (0–0.2 equiv.). (g) Molecular structures of GTP (as a representative guanosine nucleotide) and complex A, indicating potential coordination and hydrogen bonding sites involved in the co-assembly. (h) PXRD patterns of co-assemblies formed by complex A with GMP, GDP, and GTP.

To investigate the interaction between complex A and guanosine nucleotides (GMP, GDP, and GTP), X-ray photoelectron spectroscopy (XPS) was employed to analyze the chemical environments of key elements (Fig. 2a–c). The P 2p XPS spectra provide direct evidence of phosphate–metal interactions in the supramolecular assemblies. In both A + GMP and A + GTP systems, well-resolved doublets corresponding to P 2p_3/2_ and P 2p_1/2_ were observed, indicative of chemically distinct phosphate environments. The shift toward lower binding energies suggests enhanced coordination between phosphate groups and the Pt(ii) centers. The deconvoluted peaks at lower and higher binding energies were assigned to the coordinated P–O–Pt species and the non-coordinated (or hydrogen-bonded) P=O groups, respectively. This behavior arises from distinct spatial arrangements of the nucleotides: GMP inserts fully between molecular layers, allowing uniform Pt(ii) coordination, whereas the longer GTP adopts a twisted conformation with its guanine and ribose moiety excluded from the Pt-stacked region. This spatial orientation reduces steric hindrance and enables its phosphate groups to interact with the Pt center same as GMP. In contrast, the A + GDP assembly showed a single broadened P 2p peak, likely due to its intermediate molecular length placing the phosphate moieties near the layer edge, where steric hindrance limits effective coordination and yields electronically averaged environments. Notably, the stronger coordination in GMP and GTP assemblies not only stabilizes distinct phosphate states but also enhances supramolecular chirality, as the phosphate groups contribute to chiral induction, consistent with the emergence of well-defined P 2p_1/2_ features. Concurrently, the N 1s region revealed a characteristic feature consistent with guanine nitrogen coordination. The free complex A exhibited a dominant peak at ∼400.4 eV (imide N), with a minor shoulder near 399.0 eV of the C=N=C environment. Upon nucleotide binding, a pronounced increase at ∼399.0 eV and a shift in the higher binding energy peak were observed, suggesting electron donation from the guanine nitrogen to the Pt(ii) center. The progressive enhancement of this signal from GMP to GTP supports a multidentate interaction model, where both phosphate and nucleobase nitrogen atoms participate in binding. Moreover, the appearance of a new O 1s peak at 533.0–534.0 eV, attributed to the carbonyl oxygen of guanine, supports the existence of π–π interactions between the guanine moiety and the aromatic ligand of complex A. The whole survey spectra and other elements are shown in Fig. S21, and C 1s and Pt 4f signals collectively suggest that both the phosphate group and guanine nitrogen atoms play key roles in whole interaction with complex A.^13^

To further identify the specific reactive sites within the nucleotides, NMR titration experiments were conducted (Fig. 2d–f). The ^1^H NMR spectrum of complex A exhibited a broad peak in the range of 6.5–7.5 ppm, suggesting the occurrence of self-assembly. This broadening is attributed to the exchange of protons among the four aromatic rings, indicating the presence of strong π–π stacking interactions and Pt⋯Pt interactions. In contrast, the signal corresponding to Ha (as shown in the molecular structure in Fig. 2g) on the pyridine ring appeared as a distinct and sharp peak, demonstrating that the core structure of complex A remained intact in the assembled state. Upon incremental addition of GMP (0.02–0.2 eq.) to complex A (Fig. 2d), significant upfield shifts were observed for the proton signals of GMP, particularly for H_1_, H_2_, H_3_, and H_4_ (4.7–5.2 ppm), H_5_ (6.6 ppm), and H_6_ (7.9 ppm), which contribute to non-covalent π–π binding between the phenyl ring of complex A and ribose or guanine. The interaction mechanism between GDP and complex A (Fig. 2e) follows a similar pattern to GMP. However, due to the increase in phosphate groups, the overall molecular length increases and the driven proton transfer of the base moiety weakens, which are reflected in the broadened and less intense signals of H_5_ and H_6_. Meanwhile, when GTP was added to complex A (Fig. 2f), a distinct broadening of the signals corresponding to H_1_, H_2_, H_3_, and H_4_ was observed, indicating that these protons participated in molecular aggregation. This broadening effect suggests that GTP enhances the overall assembly, leading to a more compact supramolecular packing.^14^ Combined evidence from NMR titration and XPS analysis reveals that the assembly of guanosine nucleotides with complex A is primarily driven by three types of noncovalent interactions: electrostatic interaction between the phosphate groups and the cationic Pt center, π–π stacking involving the ribose ring and aromatic ligands, and weak coordination between guanine nitrogen atoms and the ligand. These synergistic interactions govern the stability and chiral organization of the resulting supramolecular structures.

To elucidate the distinct assembly behaviors of complex A with the three guanosine nucleotides, powder X-ray diffraction (PXRD) analysis was conducted on the samples before and after mixing. The PXRD pattern of complex A exhibits five sharp diffraction peaks at 2*θ* = 6.7°, 11.5°, 13.3°, 17.6°, and 20.0°, corresponding to the (100), (110), (200), (210), and (300) crystal planes, respectively (Fig. S22A). These reflections confirm that complex A adopts a 2D hexagonal lattice structure with a lattice parameter of *a* = 1.32 nm, as determined from PXRD analysis. Additionally, a diffraction peak at 2*θ* = 26° (*d* = 0.38 nm) indicates the presence of Pt⋯Pt or π–π stacking interactions within the self-assembled structure, consistent with a Pt⋯Pt distance of 3.38 Å^10*a*^ (Fig. S23). Among the guanosine nucleotides, GMP exhibits pronounced diffraction peaks, indicating its high crystallinity, while GDP and GTP display amorphous characteristics due to the presence of multiple phosphate groups, which induce stronger electrostatic repulsion and greater molecular flexibility (Fig. S22B). Upon reacting with complex A, the (100) diffraction peak shifts to lower angles, with the interlayer spacing increasing from 1.28 nm to 1.50 nm. This shift can be attributed to the intercalation of nucleotide molecules into the interlayer region of complex A through electrostatic interactions, leading to expanded interlayer spacing (Fig. 2h). Furthermore, the low-angle diffraction peaks broaden and exhibit slight splitting after nucleotide binding, a phenomenon particularly pronounced in the GDP and GTP systems. This can be ascribed to the larger molecular size and stronger interactions induced by their multi-phosphate groups, which result in structural inhomogeneity in the interlayer stacking. Notably, a new peak appears at around 2*θ* = 8.2° for both GDP and GTP systems, with interlayer spacings of 1.12 nm and 1.18 nm, respectively, consistent with the sizes of nucleotide molecules, indicating their role in generating new layered structures. Differences in phosphate group number markedly affect the molecular size of the nucleotides, resulting in notable modulation of interlayer distances and potentially driving distinct two-dimensional packing arrangements in their assemblies with complex A. Despite these structural differences, the diffraction peak near 2*θ* = 26°, attributed to Pt⋯Pt and π–π stacking interactions, exhibits a slight shift to lower angles accompanied by peak broadening upon nucleotide incorporation, suggesting a disruption of the original supramolecular order and an expansion in interlayer spacing.

### Comparative analysis of multi-optical outputs of the co-assemblies

**Fig. 3:**
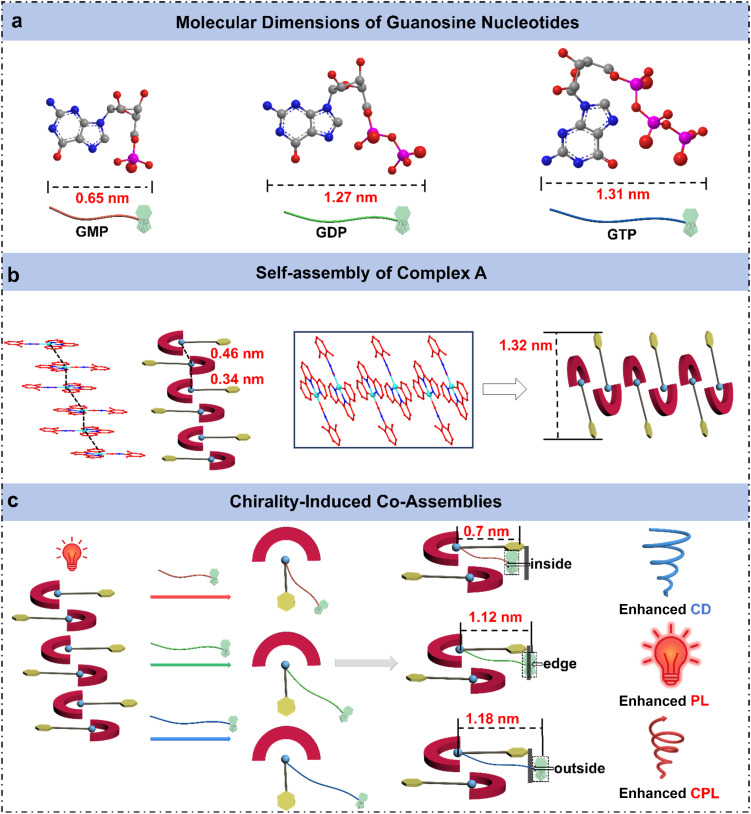
Schematic illustration of the supramolecular co-assembly mode of complex A with GMP, GDP, and GTP. (a) Van der Waals dimensions of the three guanosine nucleotides based on Bondi's atomic radii. (b) Self-assembly of complex A and the assembly model. (c) Co-assembly with GMP, GDP, and GTP yields distinct supramolecular packing modes—inside, edge, and outside insertion—leading to selective enhancement of CD, PL, and CPL, respectively.

To gain further structural insight, we calculated the van der Waals dimensions of complex A and three guanosine nucleotides based on Bondi's atomic radii (Fig. S24).^15^ The resulting size differences provide insight into their distinct supramolecular packing modes, offering a structural explanation for the different assembly behaviors observed in the three co-assemblies (Fig. 3). From a molecular geometry perspective, all three guanosine nucleotides (GMP, GDP, and GTP) adopt a bent “7-shaped” conformation, in which the phosphate group forms the tail of the structure. As the number of phosphate groups increases from GMP to GTP, the overall molecular conformation becomes increasingly curved, with the terminal phosphates bending closer toward the nucleobase. This leads to distinct molecular dimensions and spatial arrangements for each nucleotide. Upon interaction with complex A, the negatively charged phosphate groups are drawn toward the positively charged metal center *via* electrostatic interactions and insert into the interlayer space of the Pt assembly. Meanwhile, the nucleobase and ribose moieties adopt different positions depending on the molecular length and structure of the nucleotide.

In the case of GMP, the phosphate-to-base distance measures approximately ∼0.51 nm with a molecular width of ∼0.65 nm. Notably, this size is shorter than the Pt–arene spacing (∼0.7 nm) within complex A. As a result, the entire GMP molecule is capable of embedding in the Pt interlayer region. Given the slightly larger molecular width of GMP relative to the intrinsic interlayer distance (0.34–0.46 nm) (Fig. S23), the insertion of GMP leads to expansion of the Pt⋯Pt stacking distance. This is consistent with the observed shift of the (100) diffraction peak in PXRD patterns toward higher angles, indicating a larger interlayer spacing. Furthermore, the complete incorporation of the chiral nucleotide into the Pt layers enables strong chiral induction on the Pt center. Consequently, A + GMP assembly exhibits pronounced ground-state chirality, as reflected by the significantly enhanced CD signals.

With a molecular length of ∼1.27 nm, GDP coordinates to the Pt center while allowing its nucleobase moiety to extend beyond the molecular layer. This configuration leads to the formation of a new crystal plane, as evidenced by the emergence of a diffraction peak corresponding to an interlayer spacing of 1.12 nm. Consequently, in the A + GDP assembly, the nucleobase moiety of GDP is situated near the edge of the molecular layer, where steric hindrance restricts molecular motion and suppresses non-radiative decay pathways, thereby enhancing the photoluminescence. However, the inferior molecular length spatially limits chirality transfer, resulting in weaker CD and CPL signals. As the longest nucleotide (∼1.31 nm), GTP adopts an extended conformation that leads to the full exclusion of its nucleobase and ribose units from the Pt⋯Pt interlayer region. This distinct packing mode induces the formation of a new layered structure, as evidenced by the emergence of a diffraction peak at *d* = 1.18 nm in the PXRD data. Steric confinement imposed by the intercalation of GDP and GTP molecules within the complex A packing structure during co-assembly can result in molecular distortion and slight variations in length, accounting for the appearance of additional peaks in the PXRD pattern. Remarkably, although GTP's chiral group does not significantly perturb the ground-state structure (hence showing weak CD), its exposed positioning facilitates efficient excited-state chirality transfer, yielding the most intense CPL signal. These findings unveil how subtle variations in phosphate group number among guanosine nucleotides translate into pronounced differences in molecular conformation and spatial dimensions, which in turn dictate the packing geometry, supramolecular chirality, and optical output. This mechanistic understanding offers valuable insights into the structure–function relationship in chiral co-assemblies, providing a theoretical foundation for the rational design of functional supramolecular materials.

### Mechanistic insights into multimodal optical recognition

**Fig. 4:**
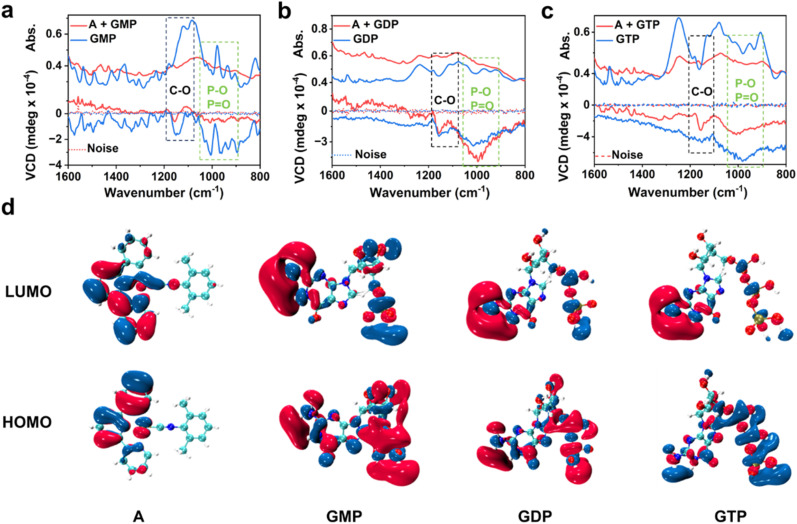
Probing chiral differences in nucleotide–complex A assemblies. IR absorption spectra (top curves) and the corresponding vibrational circular dichroism (VCD) spectra (bottom curves) of (a) GMP, (b) GDP, and (c) GTP, before and after interaction with complex A; black and green dashed boxes represent characteristic stretching vibration absorption of C–O and P–O/P=O, respectively, highlighting the chiral source in these vibrational regions. (d) HOMO and LUMO frontier orbital electron density distributions of complex A, GMP, GDP, and GTP. Red and blue isosurfaces represent positive and negative wave functions, respectively. The distributions of the highest occupied molecular orbital (HOMO) and lowest unoccupied molecular orbital (LUMO) reveal the electronic properties of each component in intermolecular interactions.

To obtain deeper insight into the chiral induction mechanism within the co-assemblies of complex A and guanosine-based nucleotides, detailed analyses of vibrational circular dichroism (VCD) spectra were conducted (Fig. 4a–c), which demonstrate ground-state chiral induction patterns that align with their different stretching vibrations. For the A + GMP assembly, a strong pair of opposite-sign VCD signals is observed in the 1170–1120 cm^−1^ region, corresponding to the stretching vibrations of the C–O groups in the ribose unit. The pronounced signal indicates that the chiral center lies in close proximity to the Pt(ii) core, resulting in strong ground-state chiral induction and a significantly enhanced CD response. In comparison, the A + GDP and A + GTP assemblies show weaker or single-sign features in the C–O region but exhibit broader negative VCD bands in the 1000–900 cm^−1^ range, associated with the stretching vibrations of phosphate (P=O and P–O) groups. These results suggest that GDP and GTP induce chirality primarily *via* interactions involving their extended phosphate chains, rather than through direct ribose coordination, and as a consequence the resulting ground-state chiral induction is notably weaker compared to that observed for A + GMP.^16^

Furthermore, to better understand the origins of chiral induction and its correlation with the observed optical responses, we analyzed the HOMO and LUMO electron density distributions of individual components. As shown in Fig. 4d, the LUMO of complex A is mainly localized over its conjugated aromatic ligands and metal center, indicating a strong π-electron density associated with the Pt(ii) chromophore. The HOMO is also largely delocalized across the ligand framework, suggesting that complex A itself is capable of participating in efficient π–π stacking and potential charge-transfer interactions upon supramolecular assembly. In GMP, the LUMO is distributed predominantly over the guanine moiety, while the HOMO spans the guanine base and partially the ribose unit, reflecting a compact electronic structure centered around the chiral core. This localized orbital distribution implies that upon co-assembly with complex A, the ribose and guanine bases are positioned in close proximity to the metal center, facilitating strong local chiral induction. This explains the distinct VCD signals in the C–O region and the enhanced CD response observed experimentally for A + GMP.

In contrast, GTP exhibits a HOMO predominantly localized on the phosphate chain, particularly over the P=O and P–O moieties, whereas its LUMO is mainly distributed over the guanine base. For GDP, the HOMO shows partial localization on the guanine unit, while the LUMO similarly resides on the guanine ring. This extended delocalization of unoccupied orbitals highlights the phosphate groups as key electrophilic regions capable of engaging in long-range non-covalent interactions—such as hydrogen bonding or electrostatic contacts—with complex A. These features help rationalize the broad VCD bands observed in the phosphate stretching region for the A + GDP and A + GTP systems. The greater number of phosphate groups enhances the polarizability and potential for supramolecular interaction, enabling a more distributed chiral environment even without strong ribose–metal coordination.

Collectively, the combined analysis of VCD spectra and frontier molecular orbitals offers a coherent mechanistic understanding of the multi-channel chiroptical responses observed in the supramolecular assemblies. The pronounced paired VCD signals in the ribose-associated C–O stretching region for GMP, together with its compact HOMO localization on the guanine base and ribose unit, point to strong local chiral induction near the Pt(ii) center, consistent with the significantly enhanced CD response. In contrast, GTP exhibits broadened VCD bands in the phosphate stretching region and displays markedly extended HOMO delocalization over the triphosphate chains. These features suggest that long-range phosphate-mediated interactions dominate chiral perturbation in this system, accounting for the intense CPL signal in A + GTP. These findings highlight how structural variations in nucleotide phosphates dictate the spatial origin and nature of chiral induction—whether localized or extended—thereby modulating the optical activity across ground and excited states in a mode-selective manner.^12,17^ These insights provide a solid foundation for the rational design of chiroptical materials based on nucleotide-mediated co-assembly, enabling advanced applications in multimodal molecular recognition, signal discrepancy, and intelligent sensing platforms.

## Conclusions

We present a structure-guided recognition strategy in which the number of phosphate groups in GMP, GDP, and GTP direct different binding geometries with organoplatinum complex A, yielding multimodal optical discrimination. GMP, bearing a single phosphate, undergoes full intercalation that positions its chiral center in close proximity to the metal core, resulting in the strongest CD signal through efficient chirality transfer. In contrast, GDP adopts a partially intercalated configuration near the edge of the molecular layer, which restricts its rotational freedom and suppresses non-radiative transition, leading to enhanced phosphorescence. While GTP forms a spatially separated assembly, wherein the chiral guanine and ribose units are positioned outside the layered structure, facilitating long-range electrostatic interactions that amplify excited-state chiral asymmetry and yield the most intense CPL signal. By directly linking molecular-scale electronic interactions with supramolecular organization, this work establishes a structure-dependent recognition mechanism and highlights the potential of supramolecular engineering to develop programmable, phosphate-sensitive chiral materials with tunable photonic functions.

## Author contributions

J. X., F. Z., and Z.-W. L. conceived the project. J. X., F. Z. and J. X. developed the methodology and performed investigations. B. Y. and J. S. handled data curation and analysis, with Z.-W. L. and P. D. contributing software. J. X. wrote the original draft, and X. J., Y. C., and Z.-Q. Y. revised it. J. X., P. D., Y. C. and Z.-Q. Y. supervised the work, acquired funding, and provided resources.

## Conflicts of interest

There are no conflicts to declare.

## Supplementary Material

SC-OLF-D5SC07250F-s001

## Data Availability

All data are available upon request. Supplementary information (SI): experimental methods and additional experimental results; Fig. S1–S24 provide complementary characterization data, including UV-vis absorption spectra, PL spectra, CD spectra, CPL spectra, XPS spectra, XRD data, *etc.*; and Table S1 summarizes the fundamental photophysical properties of organoplatinum complex A and its assemblies with various nucleotides. See DOI: https://doi.org/10.1039/d5sc07250f.
