# Supplementary material for: Guanine nucleotide directed co-assembly with Pt-complexes for tailoring chiroptical properties and multimodal molecular recognition
Source: Chem Sci. 2025 Nov 14;17(2):1330–40. doi: 10.1039/d5sc07250f (PMC12648406; doi:10.1039/d5sc07250f)
Supplement: SC-017-D5SC07250F-s001 [file SC-017-D5SC07250F-s001.pdf]

Supplementary Information (SI) for Chemical Science.
This journal is © The Royal Society of Chemistry 2025

Supporting Information

Guanine Nucleotides Directed Co-assembly with Pt-Complex for Tailored Chiroptical Properties and Multimodal Molecular Recognition

Jiannan Xiao,[abc] Fang Zeng,[c] Zhi-Wang Luo,[abc] Bo Yang,[a] Jun Song,[b] Pengfei Duan,[c] Xue Jin,*[c] Yong Chen,*[d] and Zhen-Qiang Yu*[a]

[a] College of Chemistry and Environmental Engineering, Shenzhen University, Shenzhen 518060, China

[b] College of Physics and Optoelectronic Engineering, Shenzhen University, Shenzhen 518060, China

[c] Laboratory of Nanosystem and Hierarchical Fabrication, National Center for Nanoscience and Technology, Beijing 100190, China

[d] Technical Institute of Physics and Chemistry & University of Chinese Academy of Sciences, Beijing 100190, China

E-mail: zqyu@szu.edu.cn, jinx@nanoctr.cn, chenyong@mail.ipc.ac.cn

Experimental Method

Materials

All chemicals and solvents were purchased from commercial sources and used without further purification unless otherwise stated. Milli-Q ultrapure water (18.2 MΩ·cm) was used in all experiments. Guanosine 5′-monophosphate disodium salt hydrate (GMP), guanosine 5′-diphosphate sodium salt (GDP), and guanosine 5′-triphosphate trisodium salt hydrate (GTP) and others nucleotides were obtained from Sigma-Aldrich. The organoplatinum (II) complex **A** was synthesized according to previously reported procedures.[1] Solvents used for optical measurements were of spectroscopic grade and purchased from Innochem (Beijing) or Aladdin. All stock solutions were freshly prepared prior to use.

Preparation of Pt–Nucleotide Co-assemblies

To prepare the supramolecular co-assemblies, aqueous solutions of the complex **A** (1.0×10^{-3} M) and each of the 12 nucleotides (1.0×10^{-3} M) were freshly prepared using Milli-Q water. Equal volumes (200 μL each) of complex **A** and nucleotide solution were mixed in a 1.5 mL microcentrifuge tube. The mixtures were sonicated for 5 minutes to ensure homogeneous dispersion, followed by incubation at room temperature for 30 minutes to allow for complete self-assembly. The resulting uniform suspensions were either directly used for spectroscopic measurements or subjected to centrifugation and filtration to collect the solid co-assemblies. The isolated solids were vacuum-dried and used for subsequent morphological and structural characterization.

Characterization

^{1}H NMR spectra were recorded on a Bruker Avance III 400 HD spectrometer using D_2O as solvents. Fourier-transform infrared (FT-IR) spectra were obtained using a JASCO FT/IR-660 Plus spectrophotometer in KBr pellets under ambient conditions. Vibrational circular dichroism (VCD) spectra were recorded on a BioTools ChiralIR-2X VCD spectrometer using KBr cells, with sample concentrations of ca. 10–20 mg/mL. UV–visible (UV-Vis) absorption spectra were recorded on a Hitachi U-3900 spectrophotometer. Steady-state photoluminescence (PL) and time-resolved fluorescence lifetime measurements were performed on an Edinburgh FS5 and a HORIBA FluoroMax+ spectrofluorometer, respectively. Quantum yield measurements were carried out using a Hamamatsu C9920-02G integrating sphere system. Circular dichroism (CD) spectra were

measured on a JASCO J-1500 CD spectrometer, while fluorescence-detected circular dichroism (FDCD) and diffuse-reflectance circular dichroism (DRCD) spectra were acquired on the same instrument with corresponding modules. Circularly polarized luminescence (CPL) measurements were conducted on a JASCO CPL-200 spectrometer. All optical measurements were performed in deionized water (milli-q grade) at room temperature (298 k), with a sample concentration of 5×10^{-4} M. the excitation wavelength was fixed at 360 nm for all emission measurements except the concentration-dependent measurement. For photoluminescence experiments, the excitation and detection paths were arranged at a 90° angle, whereas CPL spectra were recorded in a 180° transmission geometry. Of all CD and CPL experiments, samples were loaded in 0.1 mm path length quartz cuvettes; excitation and detection axes were aligned horizontally and orthogonal to the sample surface. Linearly polarized light was used in CD measurements, while a depolarizer was used to ensure unpolarized excitation in CPL measurements.

Scanning electron microscopy (SEM) images were obtained on a Hitachi S-4800 field-emission SEM at an accelerating voltage of 10 kV. Prior to imaging, samples were deposited on silicon wafers and sputter-coated with a thin layer of platinum to enhance contrast. Transmission electron microscopy (TEM) was performed on a JEOL JEM-2100 microscope operating at 200 kV. X-ray photoelectron spectroscopy (XPS) was carried out using a Thermo Scientific ESCALAB 250Xi system equipped with a monochromatic Al Kα source (1486.6 eV).

Calculation of Molecular Orbital Energy Level and Electrostatic Potential

Density functional theory (DFT) calculations were conducted to investigate the electrostatic potential surfaces and frontier molecular orbitals (HOMO and LUMO) of Complex **A** and twelve representative nucleotides. All geometry optimizations and subsequent energy evaluations were performed using the Gaussian software suite,[2] employing the B3LYP functional in combination with the 6-311G(d) basis set. For the platinum center in Complex **A**, the Stuttgart relativistic effective core potential (ECP) was applied to account for core electrons. Molecular visualizations and orbital rendering were carried out using the Visual Molecular Dynamics (VMD) program.[3]

1. Experimental results

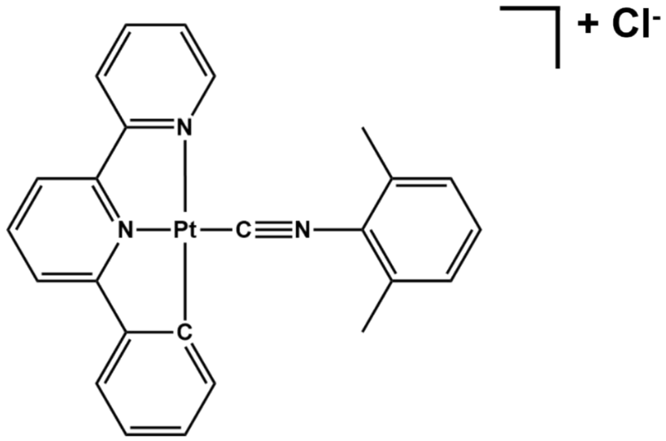

Fig. S1. The detailed structure of organoplatinum complex **A**.

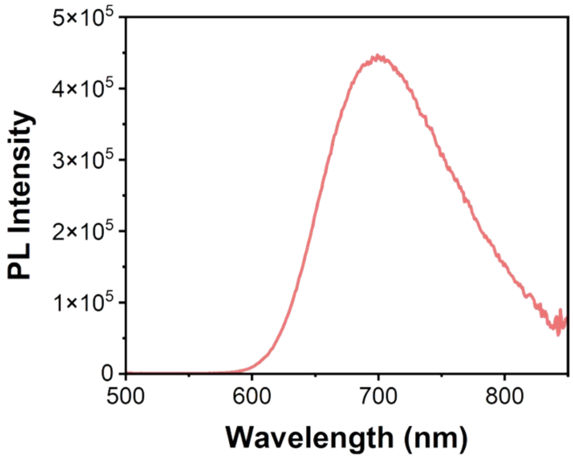

Fig. S2. Phosphorescence spectra of complex **A** in solid state under excitation at 360 nm.

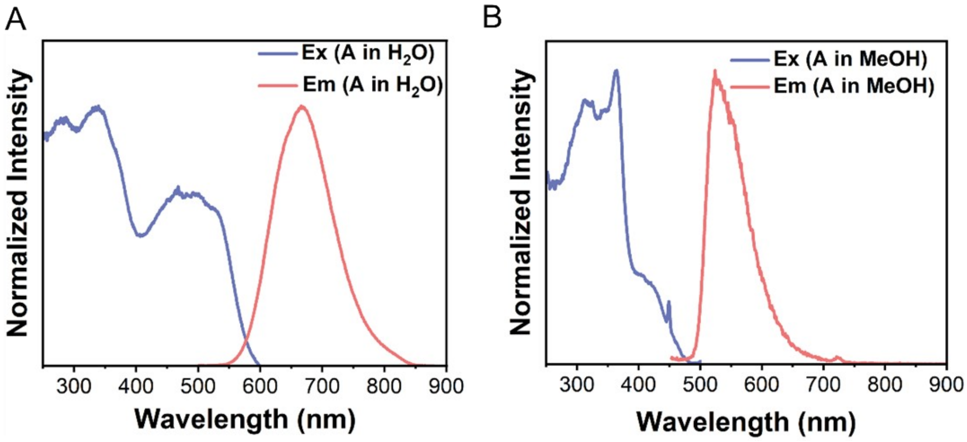

Fig. S3. The excitation and emission spectra of complex **A** in (A) H_2O and (B) MeOH solution (λ_{ex} = 360 nm).

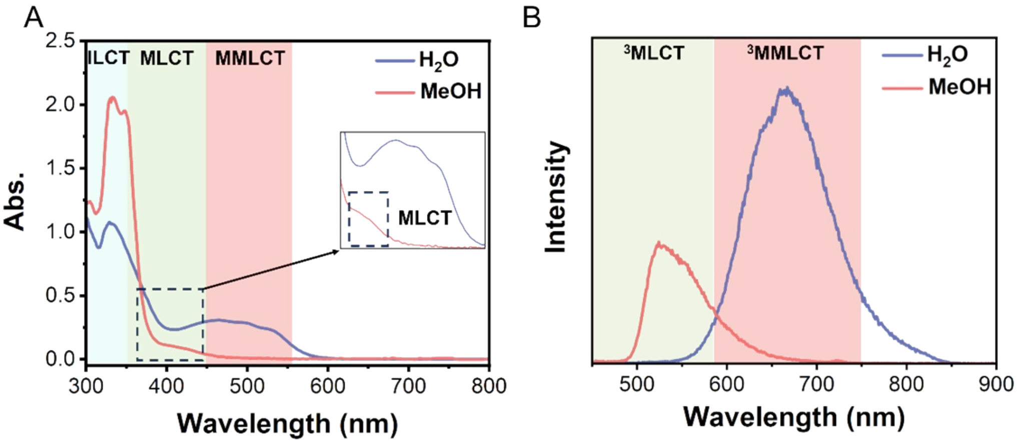

Fig. S4. The absorption spectra (A) and PL emission spectra of complex **A** in H_2O and MeOH solution (λ_{ex} = 360 nm).

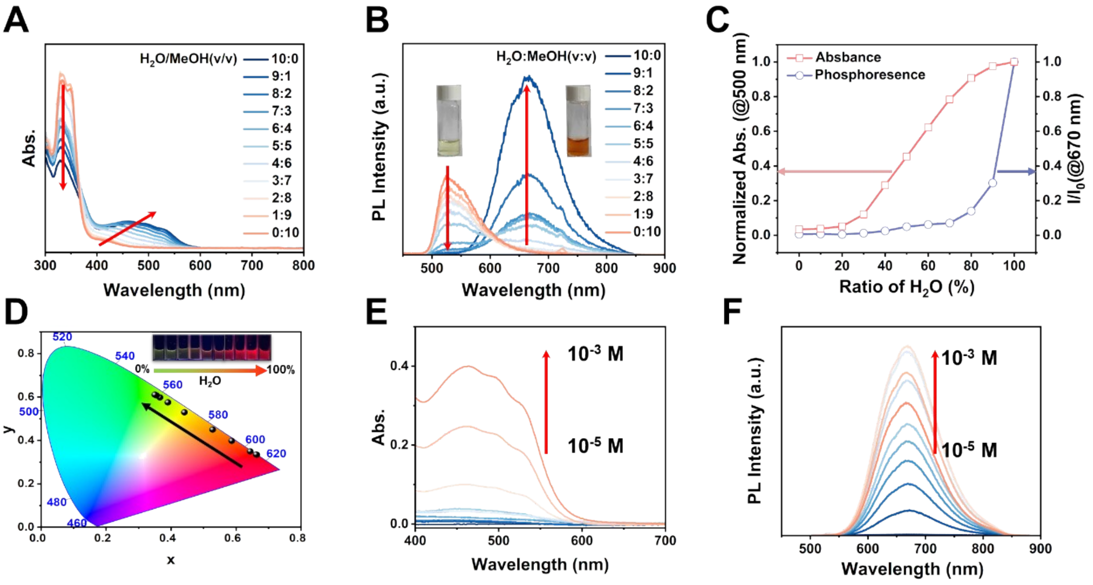

Fig. S5. (A) UV-Vis absorption spectra at different water fractions, showing aggregation-induced features (c = 5 × 10^{-4} M). (B) Corresponding PL spectra under 360 nm excitation; inset shows color change upon aggregation. (C) Normalized absorbance (red) and PL intensity (blue) versus water fraction, illustrating assembly-induced enhancement. (D) CIE 1931 chromaticity diagram showing emission color variation with water content; inset: photographs under 365 nm UV. (E) UV-Vis spectra at increasing concentrations, indicating enhanced low-energy absorption. (F) PL spectra at varying concentrations under 360 nm excitation.

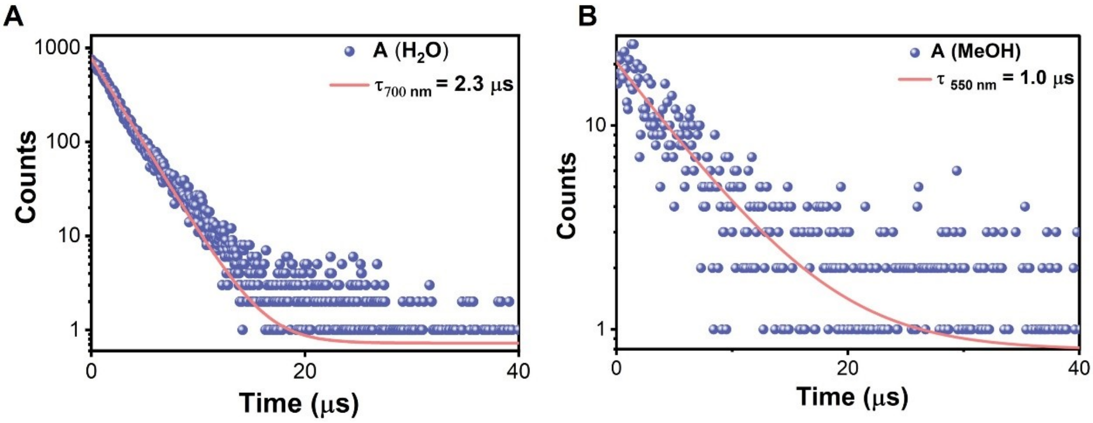

Fig. S6. Phosphorescence lifetime of complex **A** in (A) H_2O and (B) MeOH solution.

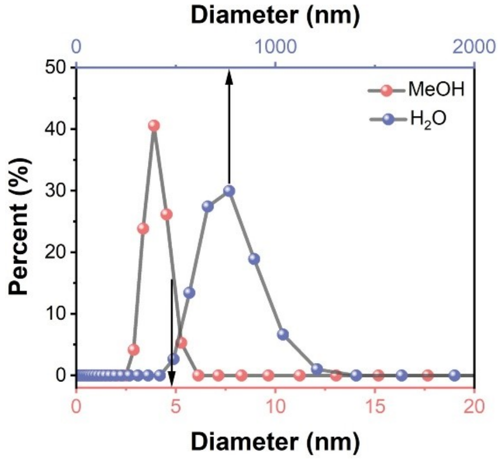

Fig. S7. DLS analysis of complex **A** in H_2O and MeOH solution.

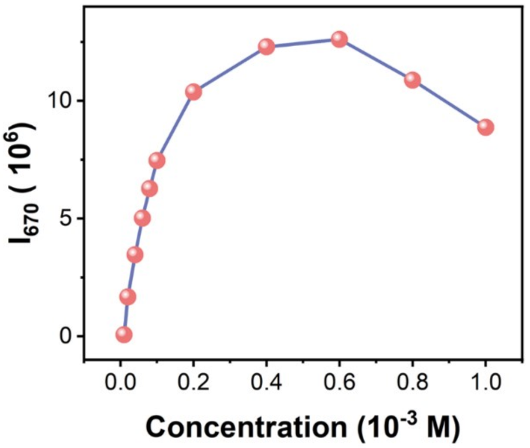

Fig. S8. The plot between phosphorescence intensity at 670 nm and concentration of complex **A**.

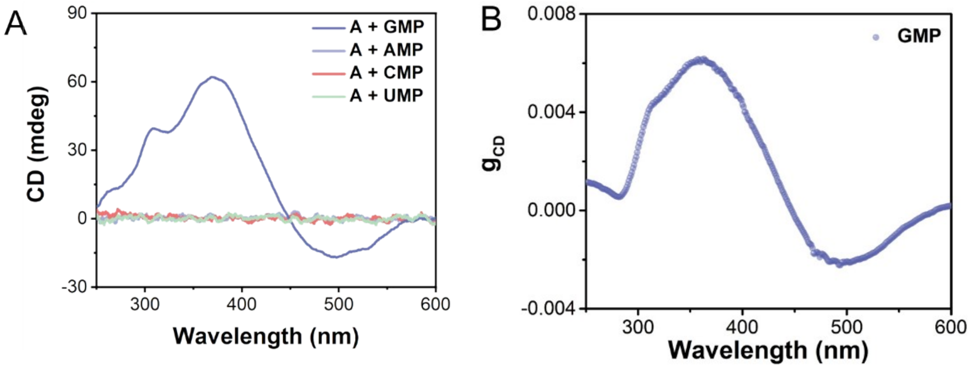

Fig. S9. (A) CD and (B) relative g_{CD} of complex **A** with others monophosphate nucleotides and GMP.

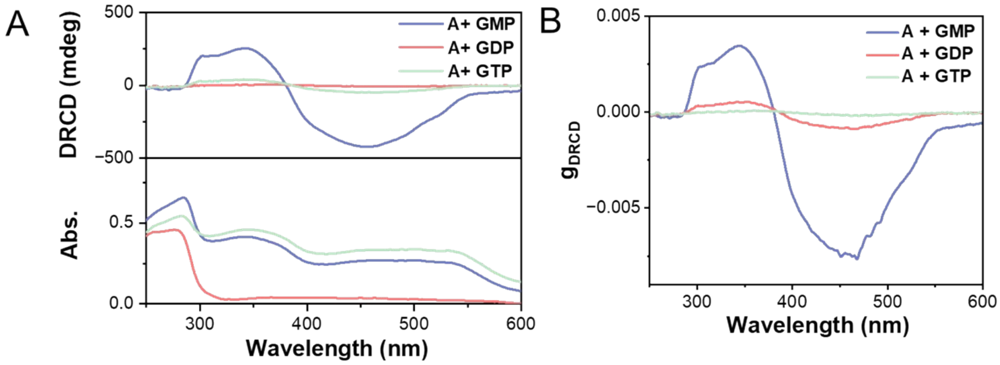

Fig. S10. (A) DRCD spectra and (B) relative g_{DRCD} of assembly **A** + GMP, **A** + GDP and **A** + GTP.

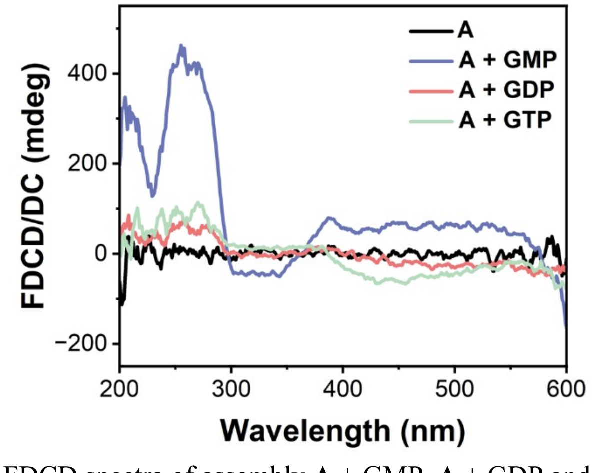

Fig. S11. FDCD spectra of assembly **A** + GMP, **A** + GDP and **A** + GTP.

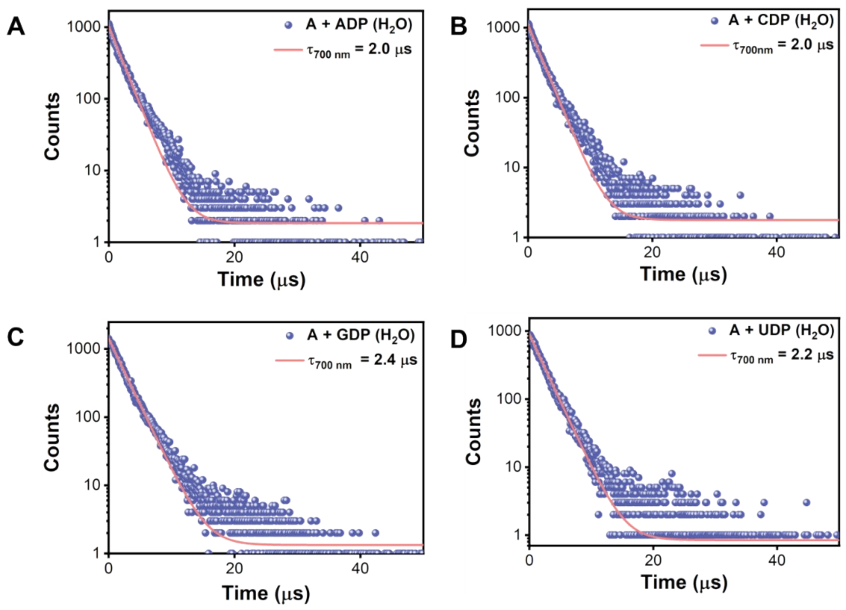

Fig. S12. Phosphorescence lifetime of complex **A** with different diphosphate nucleotides.

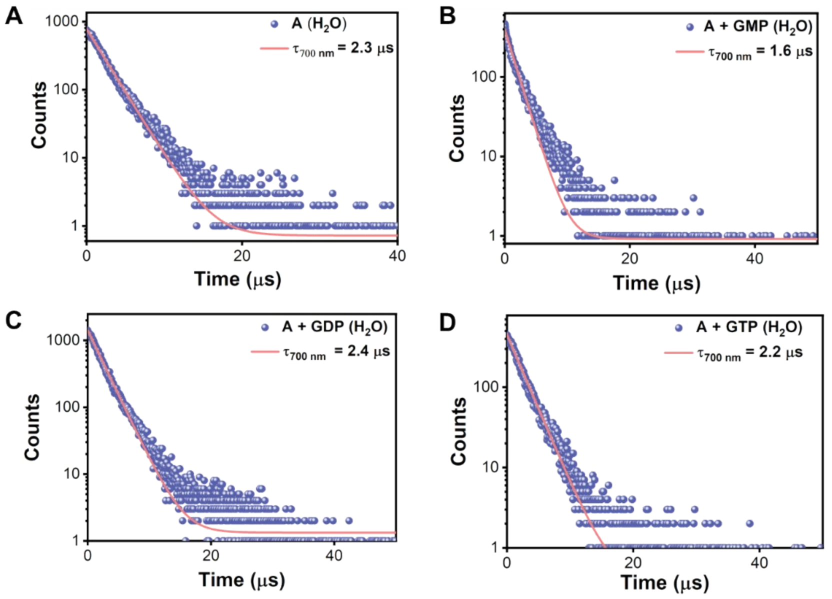

Fig. S13. Phosphorescence lifetime of complex **A** with different guanosine nucleotides.

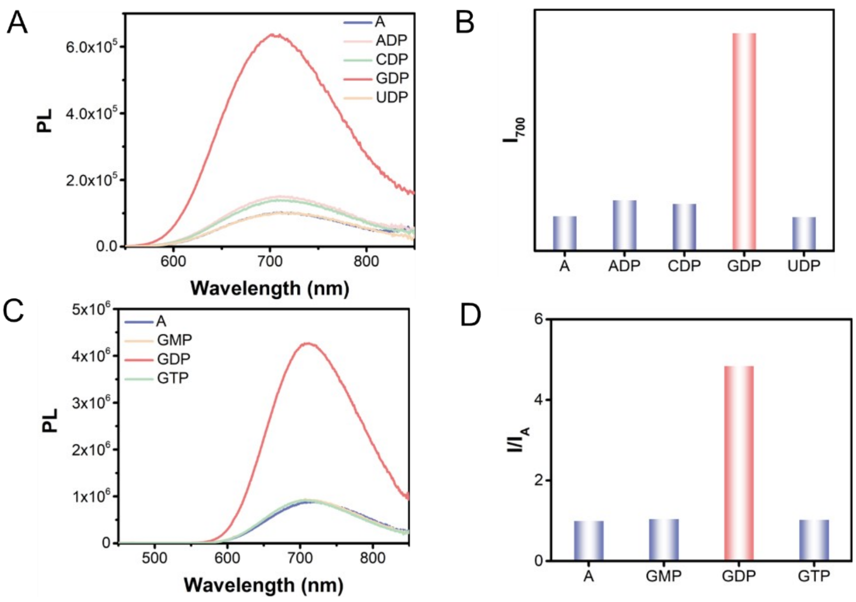

Fig. S14. Phosphorescence emission spectra of complex **A** with different guanosine nucleotides and diphosphate nucleotides (λ_{ex} = 360 nm).

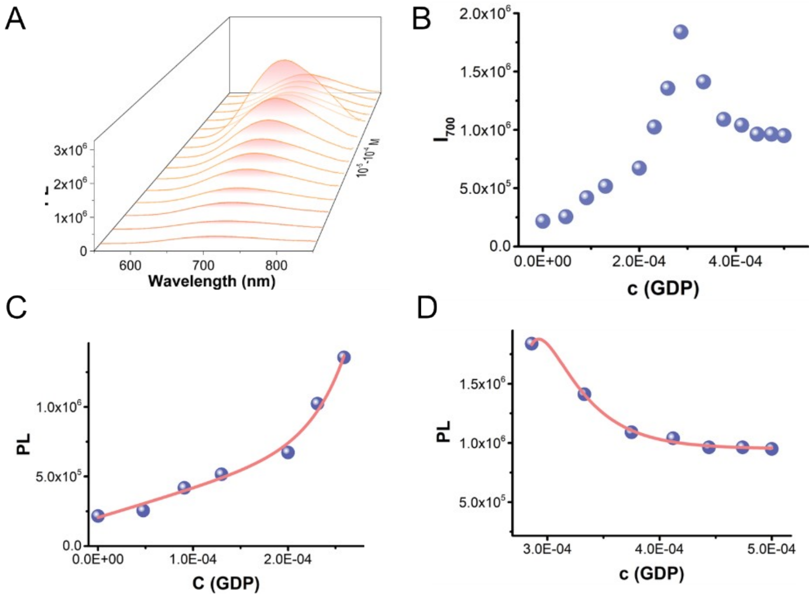

Fig. S15. Phosphorescence titration spectra of complex **A** treated by GDP.

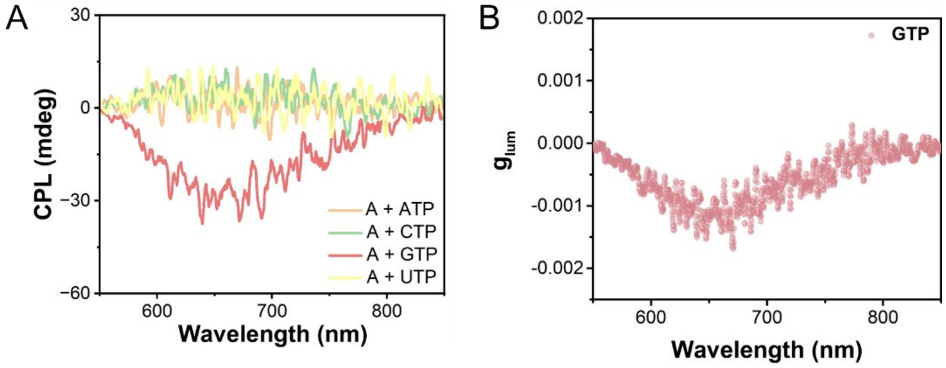

Fig. S16. (A) CPL spectra and (B) relative g_{lum} value of complex **A** with different triphosphate nucleotides and GTP.

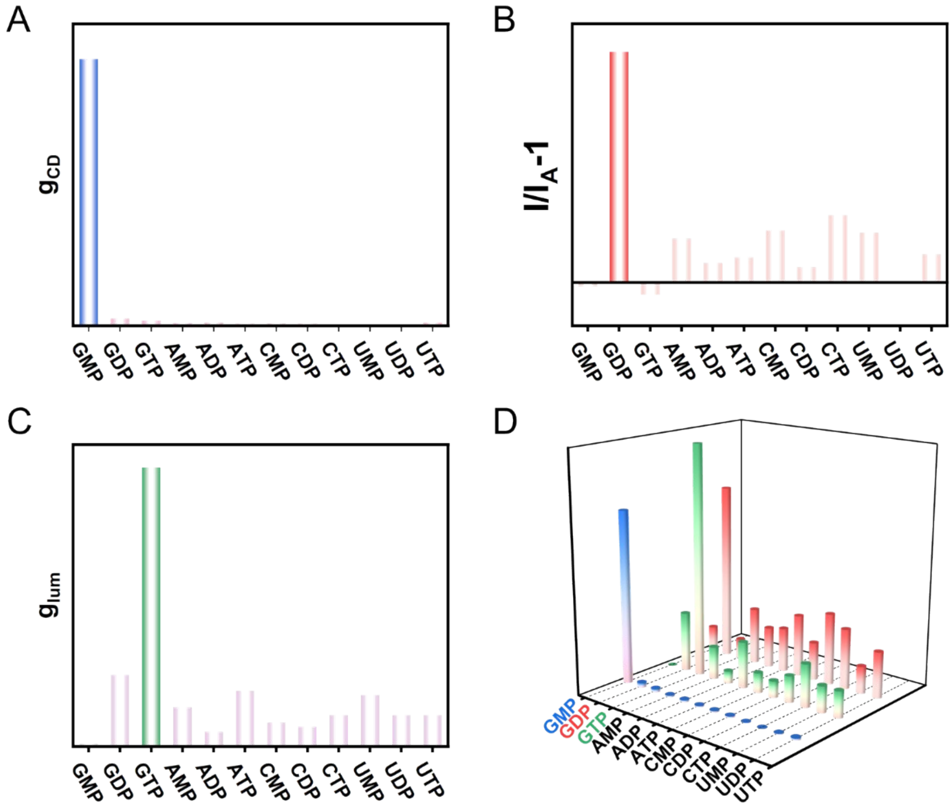

Fig. S17. (A) The column chart of $|g_{CD}|$ value, (B) relative emission intensity at 700 nm and (C) g_{lum} of CPL spectra for complex **A** with various nucleotides. (D) The 3D bar chart of distinct optical signals from **A**–nucleotide assemblies.

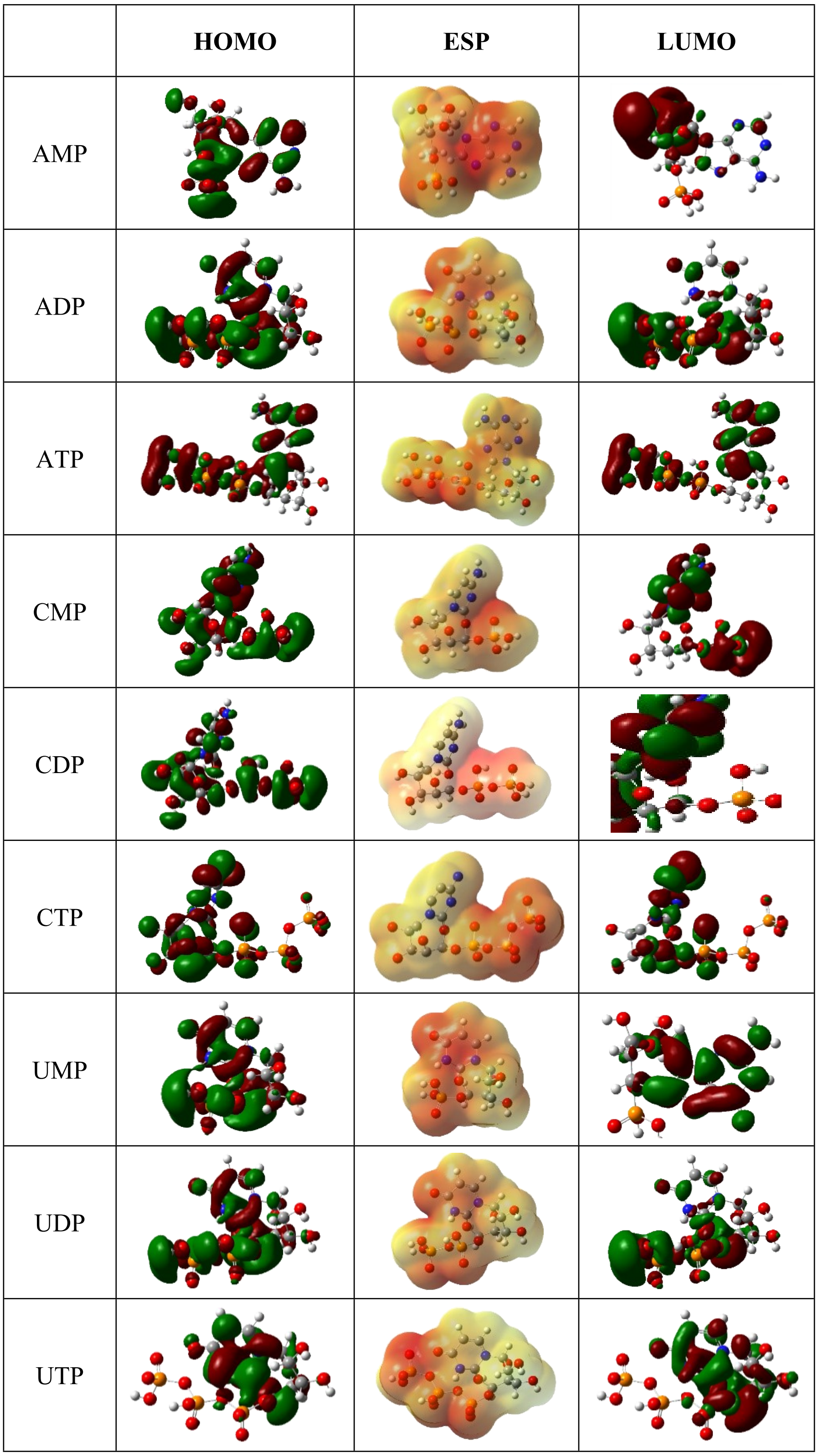

Fig. S18. HOMO/LUMO levels and relatively ESP of complex **A** and A, C, U-series nucleotides.

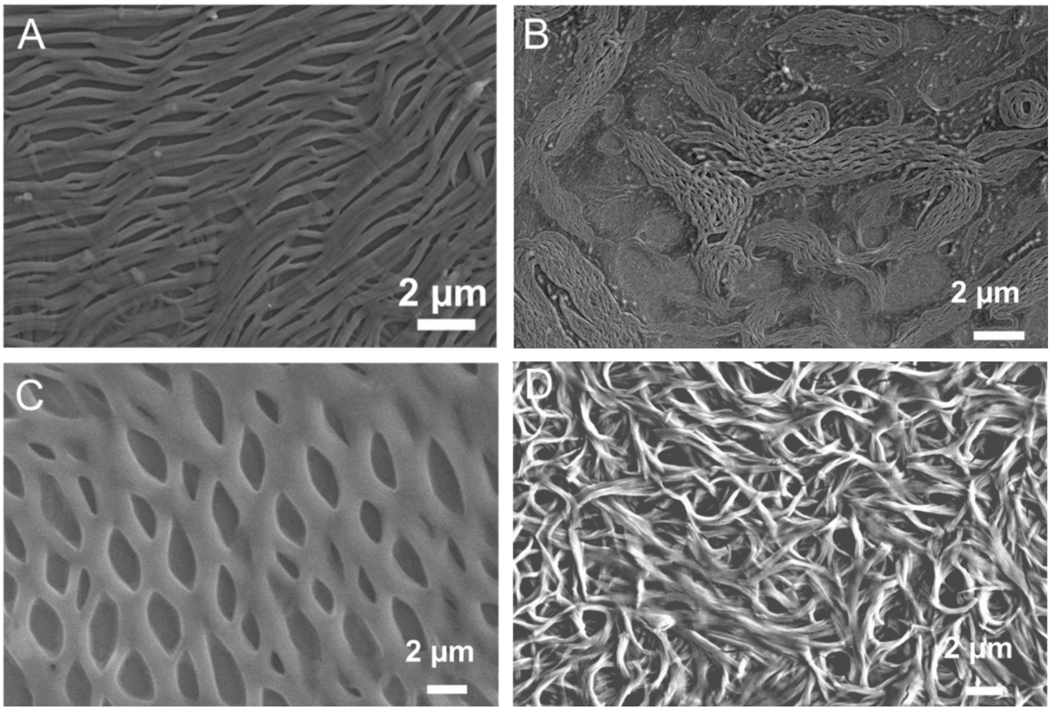

Fig. S19. (A) SEM image of complex **A** and co-assemble with (B) GMP, (C) GDP and (D) GTP.

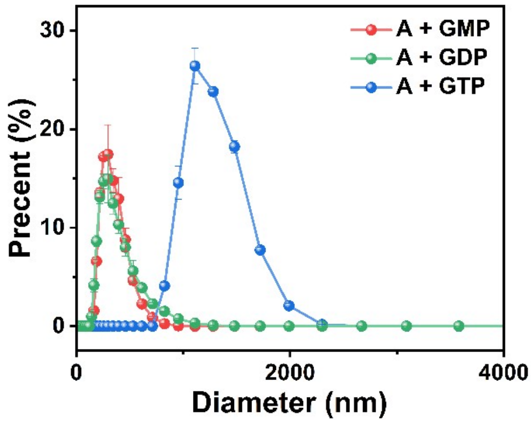

Fig. S20. DLS of assemblies of **A** + GMP, **A** + GDP and **A** + GTP.

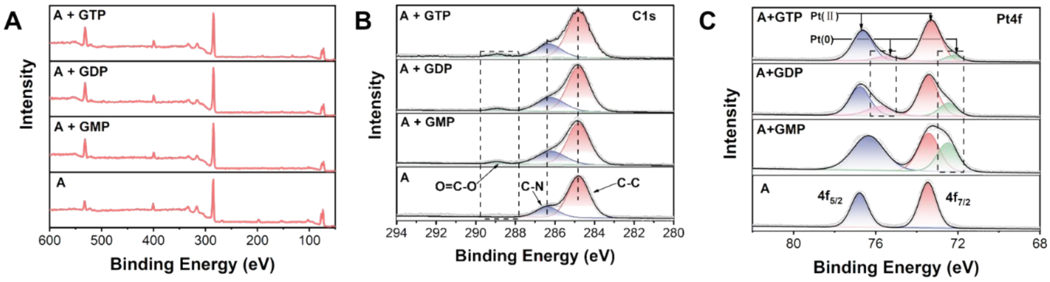

Fig. S21. (A) XPS wide-scan survey spectra, (B) C 1s, and (C) Pt 4f spectra of complex **A** and assembly **A** + GMP, **A** + GDP and **A** + GTP.

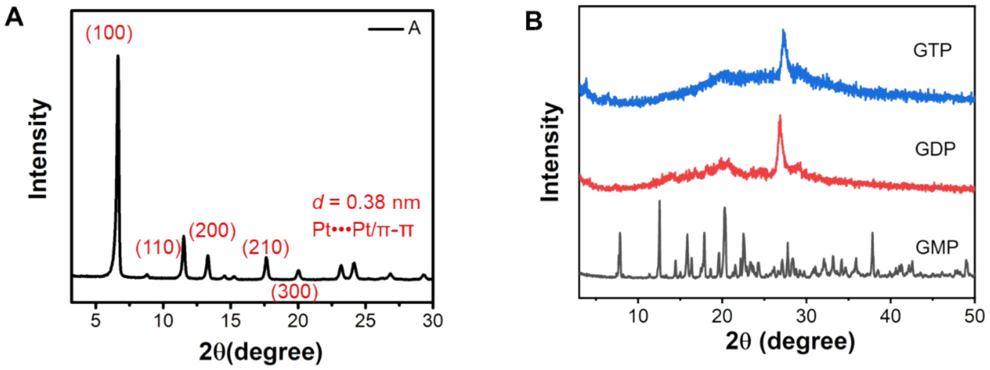

Fig. S22. (A) PXRD patterns of complex **A**, (B) pure GMP, GDP and GTP.

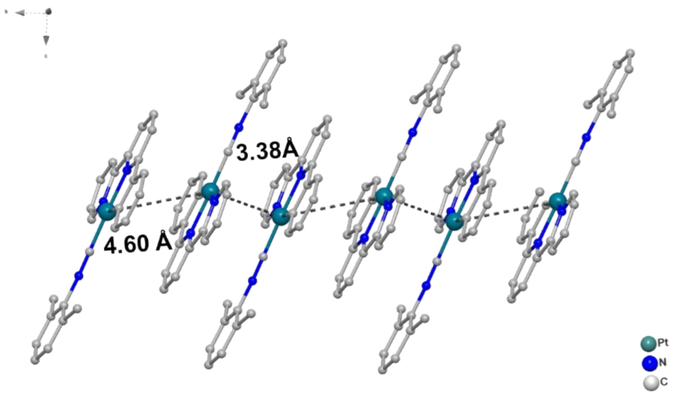

Fig. S23. Molecular packing structure of complex **A**.

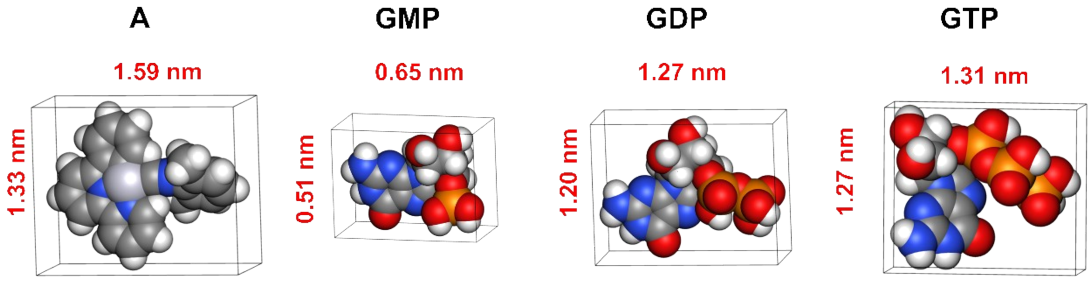

Fig. S24. Van der Waals dimensions of complex **A** and three guanosine nucleotides based on Bondi's atomic radii.

Table S1. Photoluminescence properties (at 670 nm attributed to ^{3}MMLCT) of representative sample at room temperature

sample	Quantum yield	Lifetime (μs)
**A**	24%	2.3
**A+GMP**	4%	1.6
**A+GDP**	29%	2.4
**A+GTP**	20%	2.2
**A+ADP**	8%	2.0
**A+CDP**	9%	2.0
**A+UDP**	10%	2.2

Reference

[1] W. Lu, Y. Chen, V. A, Roy, S. S, Chui, C. M, Che, Supramolecular polymers and chromonic mesophases self-organized from phosphorescent cationic organoplatinum (II) complexes in water. *Angew. Chem. Int. Ed.* **2009**, *48*, 7621-7625.

[2] M. E, Frisch, G. Trucks, H. B, Schlegel, G. Scuseria, M. Robb, J. Cheeseman, G. Scalmani, V. Barone, G. Petersson, H. Nakatsuji, Gaussian 16 Rev. C.01. 2016.

[3] W. Humphrey, A. Dalke, K. Schulten, VMD: visual molecular dynamics. *J. Mol. Graph* **1996**, *14*, 33-38.
